# The role of insect pollinators in avocado production: A global review

**DOI:** 10.1111/jen.12869

**Published:** 2021-02-09

**Authors:** Keira Dymond, Juan L. Celis‐Diez, Simon G. Potts, Brad G. Howlett, Bryony K. Willcox, Michael P. D. Garratt

**Affiliations:** ^1^ Centre for Agri‐Environmental Research School of Agriculture, Policy and Development University of Reading Reading UK; ^2^ Escuela de Agronomía Pontificia Universidad Católica de Valparaíso Quillota Chile; ^3^ The New Zealand Institute for Plant and Food Research Limited Christchurch New Zealand

**Keywords:** avocado, honeybees, insect pollination, managed pollinators, *Persea americana*, wild pollinators

## Abstract

Insect pollination increases the yield and quality of many crops and therefore, understanding the role of insect pollinators in crop production is necessary to sustainably increase yields. Avocado *Persea americana* benefits from insect pollination, however, a better understanding of the role of pollinators and their contribution to the production of this globally important crop is needed. In this study, we carried out a systematic literature review and meta‐analysis of studies investigating the pollination ecology of avocado to answer the following questions: (a) Are there any research gaps in terms of geographic location or scientific focus? (b) What is the effect of insect pollinators on avocado pollination and production? (c) Which pollinators are the most abundant and effective and how does this vary across location? (d) How can insect pollination be improved for higher yields? (e) What are the current evidence gaps and what should be the focus of future research? Research from many regions of the globe has been published, however, results showed that there is limited information from key avocado producing countries such as Mexico and the Dominican Republic. In most studies, insects were shown to contribute greatly to pollination, fruit set and yield. Honeybees *Apis mellifera* were important pollinators in many regions due to their efficiency and high abundance, however, many wild pollinators also visited avocado flowers and were the most frequent visitors in over 50% of studies. This study also highlighted the effectiveness of stingless bees (Meliponini) and blow flies (Calliphoridae) as avocado pollinators although, for the majority of flower visitors, there is a lack of data on pollinator efficiency. For optimal yields, growers should ensure a sufficient abundance of pollinators in their orchards either through increasing honeybee hive density or, for a more sustainable approach, by managing wild pollinators through practices that protect or promote natural habitat.

## 1 INTRODUCTION

Avocado is one of at least 105 crops that receive yield benefits from animal pollination (Rader et al., 2020), and together, these crops represent approximately 35% of total agricultural production (Klein et al., 2007). Insects are the most important animal pollinator and therefore, to sustainably increase food production and feed a growing population, we need to better understand the role of insect pollinators and how they can be managed effectively in important animal‐pollinated crops such as avocado.

Insect pollinators are thought to facilitate avocado pollination and thus increase production, and there is evidence of opportunities to improve yield through improved pollination service. For example, under normal pollination conditions, fruit set percentage at the tree level is less than 1% whereas, with the addition of hand pollination, fruit set rates have reached 5% at the branch level (Alcaraz & Hormaza, 2009; Evans et al., 2010; Garner & Lovatt, 2008). Furthermore, like many insect‐pollinated crops, avocado yields may be adversely affected by widespread pollinator declines (Biesmeijer et al., 2006; Potts et al., 2016).

Optimizing avocado yields is increasingly important as demand for this product is rising with 32.6 million tonnes produced from 1999 to 2008 and 50.4 million tonnes from 2009 to 2018 globally (FAO, 2020). Today, avocados are not only a nutritious staple but also an important export crop for many countries (USD 6.84 billion globally for 2018) (FAO, 2020). However, in some avocado growing regions, expansion is having adverse environmental impacts such as, biodiversity loss and water resource depletion (Magrach & Sanz, 2020) and thus improving sustainable production is crucial.

Avocados have a synchronous, dichogamous flowering pattern. Flowers are hermaphroditic (have both male and female parts) but open as female and male separately at different times and this differs between cultivars. In ‘A‐type’ cultivars, flowers commonly open as functionally female in the morning of the first day and functionally male in the afternoon of the second day, whereas, in ‘B‐type’ cultivars, flowers are commonly female in the afternoon of the first day and male in the morning of the second day (Nirody, 1922; Stout, 1932). This process encourages outcrossing, however, avocados are self‐fertile and pollination from within the same cultivar or tree (close pollination) can occur during the daily overlap of male and female flowers (Nirody, 1922; Stout, 1932). Daily overlapping is a common occurrence, but weather conditions play an important role in flowering synchronization and, under cooler temperatures, the length of time for male and female flowers to overlap can significantly increase (Ish‐Am & Eisikowitch, 1990; Pattemore et al., 2018). In theory, self‐pollination is possible during the male opening, as stigmas can still be receptive (Davenport et al., 1994), however, successful fertilization during this phase is extremely rare (Sedgley, 1977; Sedgley & Grant, 1983).

The dichogamous flowering pattern and other aspects of the avocado flower morphology (e.g. heavy and large pollen grains, the release of a low number of pollen grains, the production of nectar, and the small stigma size), indicate a probable important role for insect pollinators in avocado pollination (Dafni, 1992; Gazit & Degani, 2002; Sedgley & Griffin, 1989; Stout, 1932; Vithanage, 1990). Additionally, several studies have explored the effect of insect pollinators on avocado pollination. Controlled pollination experiments carried out by Davenport et al. (1994) and Davenport (2019) showed no significant difference in pollination rates between open‐pollinated treatments with high honeybee hive density compared with closed pollination treatments with no access to insect pollinators; therefore, it is argued that wind pollination is the dominant pollination mechanism in these systems. However, most other studies have shown that without insect pollinators; pollination (Cabezas & Cuevas, 2007), fruit set (Can‐Alonzo et al., 2005; Malerbo‐Souza et al., 2000) and yields (Mulwa, Kahuthia‐Gathu, et al., 2019; Petersen, 1955; Robbertse & Johannsmeier, 1997) are significantly reduced in comparison with open‐pollinated treatments. A better understanding of the role of insect pollinators in avocado production and what factors result in this variation is needed.

Additionally, across the globe, there is growing evidence of the contribution from wild pollinators and natural habitats to pollination services (Dainese et al., 2019; Martin et al., 2019; Garibaldi et al., 2011, 2013). It is therefore important to determine which pollinators are pollinating different crops, including avocado, and identify effective ways to improve and protect this ecosystem service. A review of avocado pollination ecology is necessary to inform sustainable management of this important ecosystem services as well as to help target future research.

We build on previous reviews on avocado pollination and reproductive biology by Wysoki et al. (2002), Gazit and Degani (2002) and Ish‐Am (2005) by providing an updated and systematic analysis of published literature on avocado pollination. The aims of this paper were as follow: (a) to consider the geographic variation and research focus of existing research on avocado pollination, (b) assess the effect of insect pollinators on avocado pollination and production, (c) identify which insect pollinators are the most abundant and effective, and how this varies by geographic location, (d) highlight potential ways to improve insect pollination for higher yields, exploring both wild and managed pollinators and (e) identify evidence gaps and direct future research.

## 2 METHODOLOGY

### 2.1 Literature review

Literature was sourced through a systematic review using the following search terms; (avocado* OR ‘Persea americana*’) AND (pollination* OR pollinators*); ‘insect pollination*’ AND (avocado* OR ‘Persea americana*’); ‘insect pollination*’ AND (avocado* OR ‘Persea americana*’) AND Management; Honeybees* AND (avocado* OR ‘Persea americana*’); ‘Pollination services*’ AND (avocado* OR ‘Persea americana*’); (avocado* OR ‘Persea americana*’) AND ‘improve pollination*’. These terms were used in two scientific databases; web of knowledge and Google scholar. In web of knowledge, all the returned searches were assessed for suitability, whereas in Google scholar, due to the high volume of searches returned, the first 500 most relevant papers were assessed. Google scholar returned a range of sources (e.g. ebooks and grey literature); however, it is possible, that this search methodology missed some wider literature. These searches provided a total of 4,043 papers in which the title and, or abstracts were assessed for suitability. Papers were selected if they had carried out original research which contributed to this review's key aims. This resulted in 35 unique papers and therefore, to increase the sample size and the range of sources, previous avocado pollination reviews were utilized to source additional relevant papers, from which a further five were found. Paper searches took place from April to June 2020. Searches were carried out in English, and if this returned a paper in another language, it was translated online and assessed for suitability. In total, the search methods produced 40 papers that were included in this review (see Table S1).

### 2.2 Data analysis

#### 2.2.1 The contribution of insect pollinators to pollination and production

A meta‐analysis was carried out to assess the difference in avocado pollination between open and closed pollinated treatments for pollination and production metrics including; the percentage of flowers pollinated, fruit set (data collected between one and three months' post‐flowering and either per branch, inflorescence or panicle), final fruit count per tree and fruit weight per avocado. The mean, standard deviation (*SD*) and sample size (*N*) were extracted directly from three papers (seven experiments); however, in many studies, this information was not provided. Therefore, studies were either excluded from the analysis or, if possible, *SD* and *N* were calculated. In one study, *SD* and *N* were calculated at the replicate level, however, other studies provided only information for different years or different orchards of differing cultivars. In these cases, year and cultivar were considered replicates, and the mean and *SD* were calculated accordingly (see Table S2). A random effects model was run using this dataset. The results showed high heterogeneity (*I* ^2^ = 95%) and therefore, an influence analysis was used to identify outliers and consequently two studies (three experiments) were removed (see Table S3 for final dataset used in meta‐analysis). This resulted in reduced, but still high heterogeneity (*I* ^2^ = 79%) and therefore, to further investigate this, the data were sub‐categorized based on plausible causes for variance including response variable (fruit set, fruit weight, pollination and yield), climate (humid or dry), cultivar (Hass or other) and experimental scale (branch, tree, site and year). To check for interdependency associated with experiments coming from the same studies, a reduced model was run with data that only included one experiment per study (see Table S4). This was used to assess if interdependency was affecting the overall result and to determine whether the full or reduced model should be used to interpret the results. All meta‐analyses models were carried out in R version 3.6.1 (R Core Team, 2019) using the ‘meta’ package (Balduzzi et al., 2019).

In addition to the meta‐analysis, to allow the inclusion of studies that had not provided all required data (*N*, *SD*, mean), a further qualitative analysis was carried out where the means of open and closed pollination treatments for each variable were summarized graphically. Data were categorized based on the response metric (fruit set per branch, per cent of flowers pollinated and fruit weight), and violin plots were created. Data which had been excluded from the meta‐analysis due to extreme outliers (as identified in the influence analysis) were also excluded from these summaries. A vote count was also implemented for controlled pollination experiments. Studies that had reported statistical significance between pollination treatments were categorized into either Open > Closed, Open < Closed or Non‐Significant and tallied. If studies had multiple but conflicting results, either from measuring different variables or applying different treatments (e.g. climate or cultivar) then an overall category was assigned based on which result was most prevalent across the different variables and treatments.

#### 2.2.2 Abundance and efficiency of insect pollinators

A qualitative assessment was also carried out to compare the relative abundance of different pollinators between studies and regions. Eighteen studies provided data on pollinator abundances and data were taken either directly from the paper or calculated from data on total observations per species. Species were categorized into broad taxonomic groups (honeybee, stingless bee, wild bees, wasp, ant, blow flies, hover flies, other diptera, coleoptera, lepidopitera, other) for comparison. Studies were then grouped by country and total abundances across all studies per country were used to calculate average country abundance. To explore pollinator efficiency, data were taken either directly from the paper or supplementary sources (see Table S5–S7). Three studies compared pollen deposition per visit, five studies explored the amount of pollen carried by pollinators, and three studies looked at the visitation rate between different groups of pollinations. A mean was calculated from the raw data and represented in box and whisker plots. All graphical summaries were produced in R version 3.6.1 (R Core Team, 2019), using the package ‘ggplot2’ (Wickham, 2016).

## 3 RESULTS

### 3.1 Research focus and geographical spread

**FIGURE 1:**
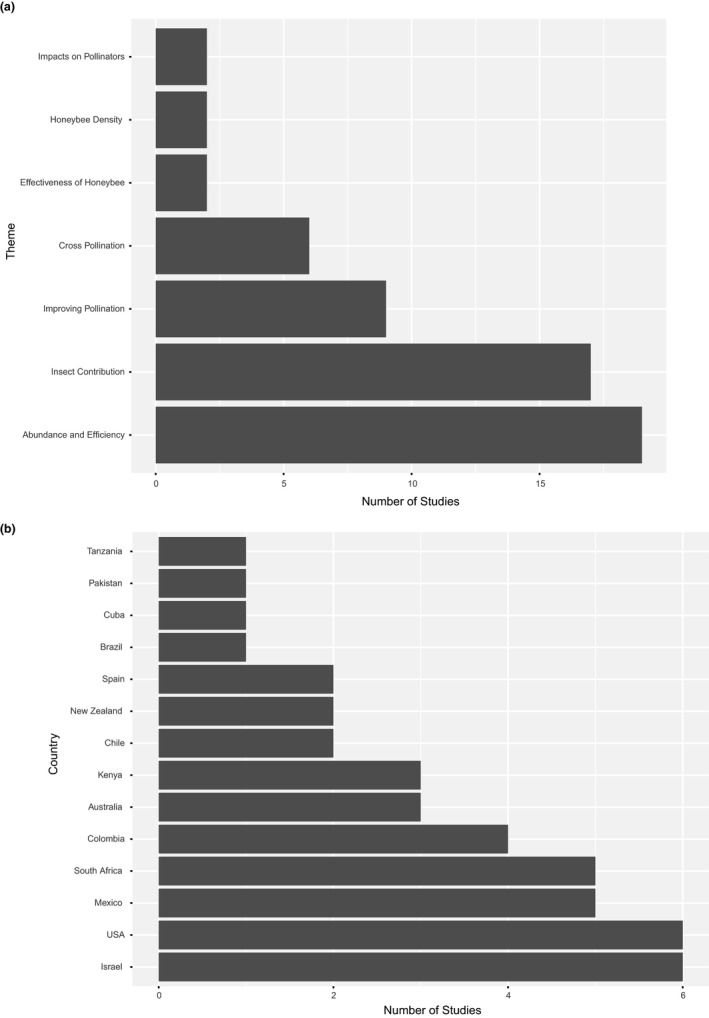
Number of studies grouped by (a) research theme and (b) country. Forty studies were used for this analysis but, multiple studies contributed to several different themes and two studies were based in two countries. (a) Research theme code: Impact on pollinators (impacts on pollinators of land management or landscape), Honeybee Density (effect of manipulating honeybee density), Effectiveness of Honeybee (effectiveness of honeybees as avocado pollinators), Improving pollination (ways to improve pollination services), Cross‐pollination (cross‐pollination contributions to pollination), Abundance and Efficiency (abundance and efficiency of avocado pollinators) and Insect Contribution (insect contribution to avocado pollination)

The majority of the studies identified by the literature search considered the abundance and efficiency of different pollinator species (33%) or the contribution of insect pollinators to pollination and production (30%) (Figure 1a). Most of the studies were carried out in the USA (14%) and Israel (14%), however, these countries count for only 6.6% of global production (data for 1999–2018) (FAO, 2020). There were less than five studies carried out per country for all remaining countries, and for three out of the top six avocado producing countries, no studies at all were identified by the search (Dominican Republic, Peru and Indonesia) (Figure 1b).

### 3.2 Contribution of insect pollinators to pollination and production

**FIGURE 2:**
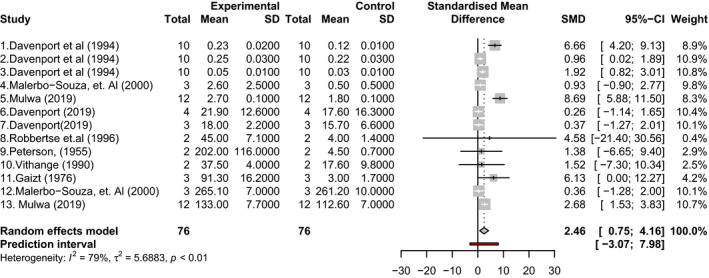
Forest plot following a random effects model meta‐analysis to compare pollination and production under insect pollination (Experimental) and no insect pollination (Control) treatments in avocado across multiple studies

**FIGURE 3:**
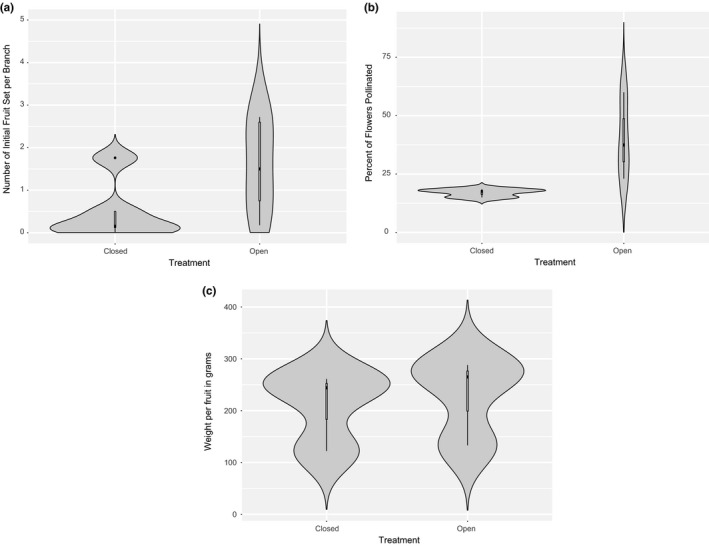
Summary means for pollination and production variables between insect pollination (Open) and no insect pollination (Closed) collected from multiple studies. (a) Average number of fruits set per branch, *N* = 5 (b) Per cent of flowers pollinated, *N* = 3 and (c) Average weight per fruit, *N* = 4

**FIGURE 4:**
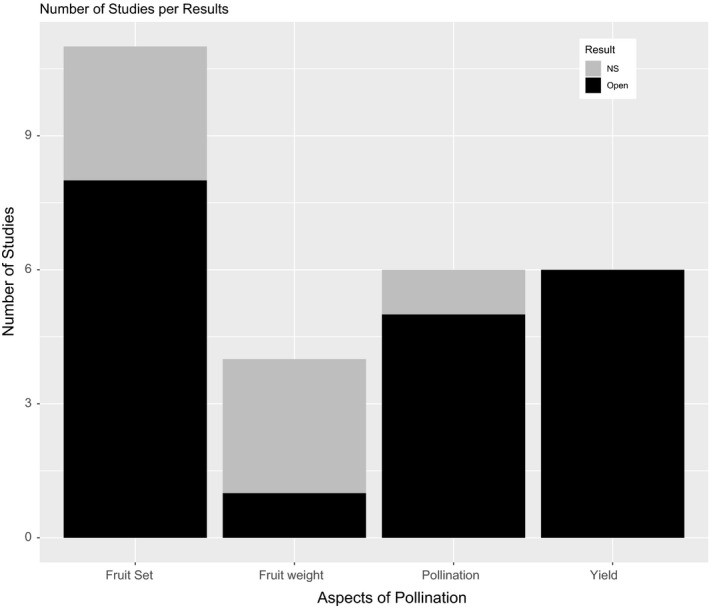
Summary of significant positive or non‐significant (NS) results following a vote count comparison across multiple studies comparing insect‐pollinated (Open) and pollinator exclusion treatments (Closed) for the variables fruit set, fruit weight, pollination and yield

Overall for the meta‐analysis, eight studies (13 data points) were included in the dataset following the removal of outliers. The result showed that over all, there was a significant increase (standardized mean difference [SMD] of 2.46 and 95% CI 0.75–4.16) in pollination, fruit set and yield in open pollination conditions compared to when insect pollinators are excluded, however, heterogeneity was high (*I* ^2^ = 79%) and significant (Figure 2). Following subcategorization by response variable, climate, cultivar and experimental scale, the SMD remained higher for open‐pollinated treatments compared to closed for all metrics considered, but heterogeneity remained high and significant for all categories indicating that variability between studies was considerable (see Figure S1). The reduced model to account for interdependency (one data point from each study) showed a similar SMD in comparison with the full model (2.7 and 2.46, respectively) and the general trend for the confidence interval was in the same direction, although this was larger in the reduced model given the fewer data points (95% CI of −0.09 to 5.49) (see Figure S2). This suggested that interdependency did not strongly affect the overall results and as such, the full model was used to interpret the results as this provided the greatest statistical power. These trends seen in the meta‐analysis were additionally supported by the outputs from the mean summaries. Nearly all key indicators showed higher values in open treatments compared with closed, and the majority of closed treatments showed close to zero pollination (Figure 3b) or fruit set (Figure 3a). There was often considerable variation in the results for the open treatments. Similarly, the findings from the vote count showed that most studies had a significantly higher value for pollination and yield in open treatments (Figure 4). Fruit weight was the only pollination variable where the majority of studies did not show a significant positive or negative effect of insect pollination (Figure 4) and the mean summaries and meta‐analysis showed only a very small difference (Figure 3c and Figure S1).

### 3.3 Abundance and efficiency of insect pollinators

**FIGURE 5:**
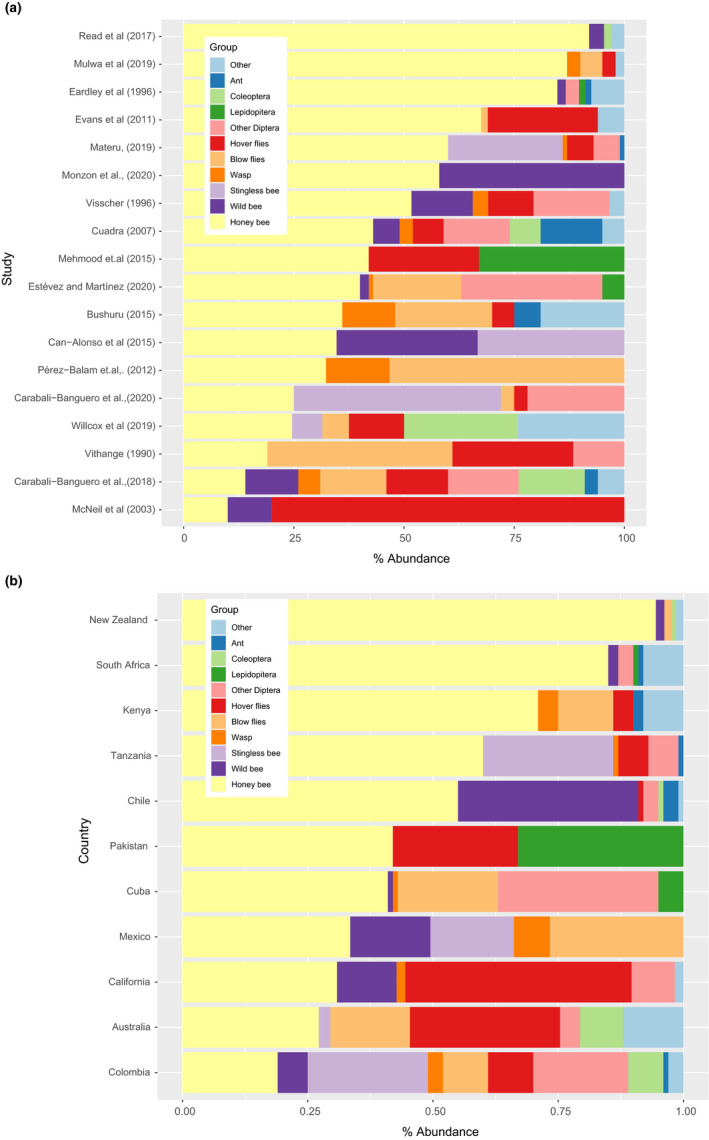
Relative abundance of pollinators visiting avocado flowers across (a) individual study and (b) grouped by country [Colour figure can be viewed at wileyonlinelibrary.com]

**FIGURE 6:**
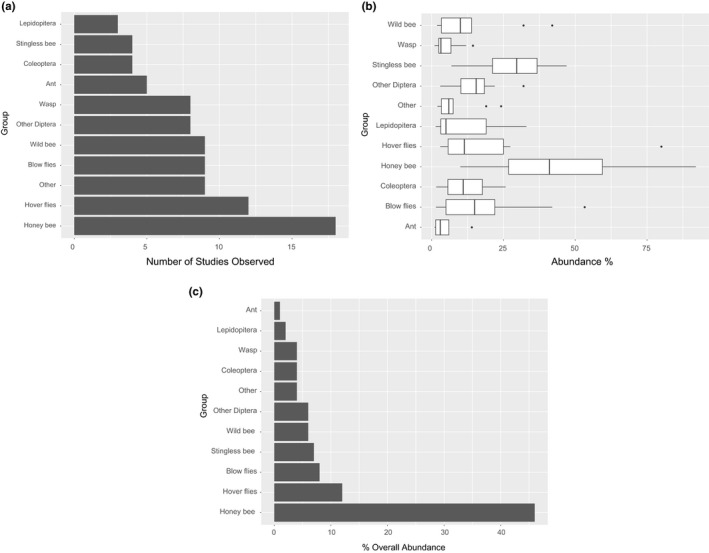
Insect groups visiting avocado flowers from 18 studies including (a) the number of studies in which each insect group was observed, (b) the total abundance of each insect group and (c) the overall relative abundance of insect groups across all studies

**FIGURE 7:**
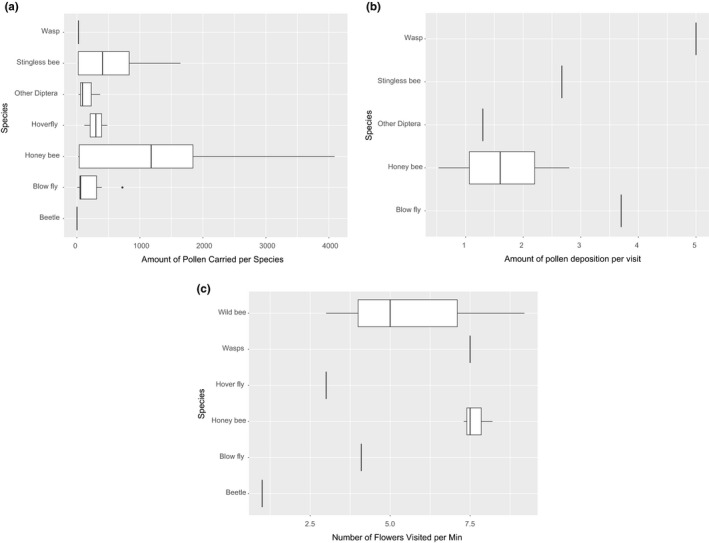
Pollination efficiency of different insect groups including (a) the amount of pollen carried per insect group, (b) the amount of pollen deposition per visit per insect group and (c) the average number of flowers visited per minute per insect group

Managed honeybees were the most frequent pollinators overall and were observed in all studies and countries (Figure 5a,b). In 13 out of 18 cases, they showed the greatest relative abundance of any single pollinator species, but this did vary considerably between 10% and 92% depending on the study. Hoverflies (Syrphidae) were also common pollinators with an overall relative abundance of 12% (Figure 6c). Stingless bees generally had high abundance in locations where they were found, but they were only observed in four studies while conversely, wild bees were seen in nine studies but had lower abundances (Figure 6a,b). Nine studies measured some aspect of pollinator efficiency but different metrics and taxa were measured and therefore, cross‐study comparisons and broader generalizations cannot be obtained from this data. However, the general trend indicates that honeybees, stingless bees, and blow flies carried and deposited the greatest amount of pollen (Figure 7a,b) and, honeybees are potentially more effective than blow flies due to the higher number of flower visits per minute (Figure 7c).

### 3.4 Improving insect pollination

Studies on improving insect pollination have generally focused on three areas; optimizing the contribution of honeybees (*n* = 4), utilizing other managed species (*n* = 4) and ways to improve pollination by wild pollinators (*n* = 2) (Table 1). The results showed that increasing honeybee density leads to significantly higher rates of pollination and production. Other managed pollinators assessed were, the New World Carniolan honeybee *Apis mellifera carnica* Pollman 1879 (*n* = 2), the buff‐tailed bumblebee *Bombus terrestris* Linnaeus 1758 (*n* = 1), and the western bumblebee *Bombus occidentalis* Greene 1858 (*n* = 1). The results suggested that buff‐tailed bumblebees could be effective pollinators, especially in the Etinger variety, as buff‐tailed bumblebee treatment plots showed higher rates of pollination and yield in comparison with honeybee treatment plots (Ish‐Am et al., 1998). The western bumblebee was shown to forage in avocado orchards and the results suggest that the introduction of bumblebee hives may increase yields; however, this study did not record bee activity on the crop (McNeil & Pidduck, 2003). Trials on the New World Carniolan honeybee explored whether this subspecies had a higher preference for avocado pollen and higher visitation rate on avocado flowers in comparison with the *Apis mellifera ligustica* Spinola 1806, however, the results showed no significant difference in visitation rate between the two subspecies (Afik et al., 2007; Fetscher et al., 2000). Two studies looked at wild pollinators and methods to increase their abundance. These studies showed that intensive management practices such as spraying pesticides and the removal of forested areas and weeds lead to a reduction in pollinator diversity (Villamil et al., 2017) and that spraying pesticides reduces wild pollinator abundance and avocado yield (Castañeda‐Vildózola et al., 1999).

**TABLE 1:** Summary of papers identified during the literature search which considered approaches to improve insect pollination and yield in avocado

Study	Main theme	Key points on improving insect pollination
Vithanage (1990)	Honeybee management	The introduction of honeybee hives during flowering led to significantly higher fruit set. Fruit size increased with beehive densities
Ish‐Am and Eisikowitch (1998)	Honeybee management	Optimal fruit set required at least five honeybees per tree per day during female flowering. Fruit set was lower when this density was not reached
Ish‐Am et al. (1998)	Other managed pollinators	Pollination rates were higher in treatments using buff‐tailed bumblebees in comparison with honeybees. In Etinger avocados, buff‐tailed bumblebees significantly increased yields whereas in Hass avocados, there was no significant difference between yields
Castañeda‐Vildózola et al. (1999)	Wild species	Many native species contributed to pollination. Spraying pesticides reduced the abundance of native pollinators and led to lower avocado yields
Fetscher et al.,(2000)	Other managed pollinators	Results showed no statistically significant difference in visitation rates between the New World Carniolan honeybees in comparison to Italian honeybees
McNeil and Pidduck (2003)	Other managed pollinators	Western bumblebees were shown to forage in avocado orchards and yields increased in rows closest to bumble hives
Afik et al. (2007)	Other managed pollinators	Trials with the New World Carniolan honeybee showed mixed results. In some locations, this subspecies had a higher avocado visitation rate than the Italian honeybee but in other locations the visitation rate was lower
Ish‐Am and Lahav (2011)	Honeybee management	There was a strong positive correlation between honeybee density and rates of pollination
Villamil et al. (2017)	Wild species	Increased forest areas, reduced spraying of pesticides and an increase of weeds in the orchard were positively associated with pollinator biodiversity
Peña and Carabalí (2018)	Honeybee management	High honeybee hive density (4 and 6 hives per hectare) resulted in higher bee density per tree and significantly higher fruit set and yield in comparison with controls (no hive)

## 4 DISCUSSION

Our review highlights the new knowledge obtained through research on avocado pollination since the last significant literature reviews were conducted more than 15 years previously. It has built on these reviews by including the analysis of 24 new studies and current topics such as the impacts of land management on wild pollinators. Additionally, we carried out a meta‐analysis on the contribution from insect pollinators to pollination and summarized global pollinator abundance and pollination efficiency of key species, thus providing a more nuanced understanding of these issues.

### 4.1 Research focus and geographical spread

The majority of studies were located in the USA and Israel. Only five studies were implemented in Mexico despite being the origin of avocados and the worlds' largest producer (30% of world production, average 1961–2018), and no studies were observed in the second‐largest producer, the Dominican Republic (7% of world production, average 1961–2018) (FAO, 2020). Further work relevant to these countries is needed as factors including local pollinator communities, climate and cultivar are likely to be unique to each region and currently, the biggest producers are not well represented. Additionally, the contribution from wild pollinators is likely higher in central American countries due to the coevolution of avocados and pollinators in this region (Brown & Cunningham, 2019; Castañeda‐Vildózola et al., 1999; Ish‐Am et al., 1999). The conclusions drawn in this review and data from other studies support this hypothesis. Studies carried out in Mexico showed that native species (e.g. stingless bees and flies) are as effective as the honeybee at avocado pollination (Can‐Alonzo et al., 2005; Castañeda‐Vildózola et al., 1999; Ish‐Am et al., 1999; Perez‐Balam et al., 2012) and furthermore, our results showed that in Mexico and Cuba, the relative abundance of wild pollinators is higher (average 63%) in comparison with other countries (average 47%). These results suggest that native pollinators in these regions may play and important role in avocado pollination and as such, the opportunity to better utilize wild pollinators for more sustainable production may be increased (Albrecht et al., 2012; Garibaldi et al., 2015; Woodcock et al., 2019). The majority of studies focused on the contribution of insects to pollination and production (16 papers) and pollinator abundance and efficiency (18 papers); however, the quality of these studies was variable. In some controlled pollination experiments, there was little or no replication and only nine of the studies on pollinator efficiency provided quantitative data.

### 4.2 Contribution of insect pollinators to pollination and production

Our findings suggest that, in most circumstances, insect pollinators make an important contribution to pollination and avocado production. The meta‐analysis and the mean summaries highlighted that there was generally a higher percentage of fruit set and flowers pollinated in open‐pollinated treatments in comparison with closed. The vote count concurred with this and similar conclusions have been drawn from other studies (excluded from this analysis due to the lack of statistical inference) (Bergh, 1967; Lesley & Bringhurst, 1951; Papademetriou, 1976) and previous avocado pollination reviews (Gazit & Degani, 2002; Ish‐Am, 2005; Wysoki et al., 2002). These findings are also supported by avocado flower morphology which strongly suggests that insects are required as pollen vectors (Dafni, 1992; Gazit & Degani, 2002; Sedgley & Griffin, 1989; Stout, 1932; Vithanage, 1990).

The results also showed a wide variation in the contribution of insect pollinators. The meta‐analysis had high and significant heterogeneity (*I* ^2^ = 79%) and, in the mean summaries, the range for open‐pollinated treatments was broad and overlapped with closed pollination treatments. A possible explanation for this variation could be that, in some circumstances, pollinators are thought to contribute little to the pollination process (Clark, 1923; Davenport, 2019; Davenport et al., 1994; Ying et al., 2009). It is hypothesized that self‐pollination is possible if stigmas remain receptive in phase 2 and this is thought to be feasible with specific cultivars (Davenport et al., 1994) and in humid climates (Gazit & Degani, 2002). Additionally, in a recent study, Davenport (2019) argues that regardless of other external factors, self‐pollination makes up a major part of avocado pollination. However, this study only measures pollen tube growth in the style and wider evidence suggests that during phase 2, pollen tubes are unable to reach the ovule and thus successful fertilization is not possible (Sedgley & Grant, 1983). Davenport (2019) also suggests that wind pollination is more prominent than insect pollination and shows that there is no difference in the per cent of pollinated stigmas between open and closed treatments. This contrasts with our findings and several other studies and thus indicates there are likely some varietal or management factors affecting the role of insect pollinators.

Variation in pollination services and resource availability between locations are probable explanations for the yield disparities in the open‐pollinated treatments. Pollinator abundance and, or efficiency, were seldom reported in controlled pollination studies and this is likely to vary significantly due to differences in; managed beehive densities, proximity to natural areas and wild pollinators, and the availability of native avocado pollinators. Variation in resource availability is also likely to be high and this has been shown to impact fruit set. Alcaraz et al. (2013) show that the amount of starch content in the style has a high correlation with the number of flowers that successfully develop into avocado fruits. Additionally, the scale at which pollination is assessed can also affect the level of contribution to pollination (Howlett et al., 2019; Webber et al., 2020) and, this may explain the significant variation between studies which had measured insect contribution at different scales (inflorescence, branch, tree).

Variation in resource availability might also explain why our results showed only limited differences in fruit weight between open and closed pollination treatments as agronomic factors that influence general tree health (e.g. irrigation), and resource availability are known to have a greater influence on fruit size (Kremer‐Köhne & Köhne, 1995). However, alternatively, this result could be an indication of pollination deficits in avocado orchards. Open‐pollinated treatments have a higher fruit set in comparison with closed pollination treatments and yet, fruit weight per avocado is similar across the two treatments. This suggests that sufficient resources are available as the tree is able to produce a good fruit weight regardless of the number of fruit set and thus, could indicate that yield deficits are the result of low pollination and reduced fruit set.

### 4.3 Abundance and efficiency of insect pollinators

Overall, managed honeybees appear to be contributing most to avocado pollination in many regions due to their general efficiency and high abundance (average overall abundance was >50%). This finding was noted in the majority of studies and has been highlighted in previous reviews (Ish‐Am, 2005; Wysoki et al., 2002). However, it is well known that honeybees can be sensitive to wind, rain and low temperatures (Bushuru, 2015; Can‐Alonzo et al., 2005) and often prefer other nectar sources (Ish‐Am & Eisikowitch, 1998). Therefore, under poor weather conditions or where other flowers are in bloom at the same time, their contribution to avocado pollination could be reduced, and diverse pollinator communities including both wild and managed pollinators may provide more consistent pollination (Woodcock et al., 2019).

Wild pollinators were also shown to play an important role. In 11 of the 18 studies and six of the 11 countries, they were more abundant than managed honeybees and although the contribution to pollination services is unknown for many wild pollinators, there is some evidence that certain wild species are efficient avocado pollinators. Stingless bees were shown to carry a comparably high amount of pollen (around 500 grains) and qualitative comments suggest they can effectively transfer pollen as their body size suits the shape of avocado flowers (Ish‐Am et al., 1999). Blow flies were also shown to be important pollinators as they deposited a high amount of pollen per visit, which may be attributed to the open structure of the avocado flower making it well suited to fly pollination (Vithanage, 1990). However, in comparison with honeybees, their flower visitation rate was low, thus reducing their potential effectiveness. The overall contribution to pollination from individual wild species is reduced due their low abundances in comparison with honeybees (e.g. the relative abundance was 28% stingless bees, 19%, for blow flies and 46% for honeybees). As such, active management to increase the abundance of certain wild pollinators may be an effective strategy to increase pollination; however, more research is required to quantify the effectiveness of wild pollinators in specific regions.

Previous studies have highlighted other potentially important pollinators. In certain locations, wasps are efficient pollinators (Ish‐Am et al., 1999; Papademetriou, 1976; Perez‐Balam et al., 2012) and a study by Ish‐Am et al. (1998) shows that managed buff‐tailed bumblebees can increase pollination. These pollinator groups were not covered in this analysis due to a lack of quantitative efficiency data and thus, this should be a target for future research. In temperate regions, it is also necessary to consider the potential of nocturnal pollinators, particularly from the diptera and lepidoptera order, as lower night temperatures can result in both male and female flowers opening during the night. Pattemore et al. (2018) showed that pollinating insects such as wood gnats, crane flies, scarab beetles, capsid bugs, forest moths and brown lacewings did visit avocado flowers during the night and were carrying avocado pollen. However, no other studies have explored this topic and therefore further investigations in this area are also needed.

### 4.4 Improving insect pollination

Tools to better manage pollination in avocado are required given the increasing production of avocados globally (FAO, 2020), the need for more sustainable production systems that make better use of inputs such as insect pollination (Garibaldi et al., 2019), and that pollination deficits in avocado are already in evidence (Alcaraz & Hormaza, 2009; Evans et al., 2010). This study highlighted three key themes to improve pollination: increasing honeybee density, utilising other managed pollinators, and exploring the potential from wild pollinators.

The results from three studies showed that increasing honeybee density led to an increase in visitation rate and an increase in pollination and production (Ish‐Am & Eisikowitch, 1998; Ish‐Am & Lahav, 2011; Peña & Carabalí, 2018). Similar findings were concluded in a meta‐analysis by Rollin and Garibaldi (2019) who showed that, for a range of insect‐pollinated crops, production does increase with honeybee density, up to a saturation point. However, for avocado production, we still lack evidence for optimal stocking densities and spatial arrangements of hives, particularly for some of the significant global producers.

Four studies focused on utilizing other managed pollinators. Trials on the New World Carniolan honeybee showed no significant difference in visitation rates between this race and the Italian honeybee (Afik et al., 2007; Fetscher et al., 2000). Conversely, a study by Ish‐Am et al. (1998) showed that buff‐tailed bumblebees were efficient pollinators, but it is thought that their high cost is currently prohibitive for wide‐scale use (Fetscher et al., 2000; Gazit & Degani, 2002) and there are risks associated with introducing managed bumblebees into countries where they are not native (Goulson, 2010; Ings et al., 2005). Stingless bees may also have the potential to be used for avocado pollination, as they are efficient pollinators and can be successfully managed (Can‐Alonzo et al., 2005; Ish‐Am et al., 1999; Quezada‐Euán et al., 2001; Ramírez et al., 2018). However, breeding on a large scale is difficult (Slaa et al., 2006) and therefore may be unfeasible for commercial systems, at least at the present time. In many countries, it may not be viable to utilize these managed pollinators and identifying and exploiting alternative pollinators may be promising. In South Africa, Eardley and Mansell (1996) suggest that increasing the abundance of carpenter bees may increase avocado pollination and research done on other crops has highlighted the contribution to pollination services from drone flies *Eristalis tenax* Linnaeus 1758 (Howlett & Gee, 2019) and blow flies (Cook et al., 2020). These studies suggest the potential for managing a range of different pollinators, but more research is needed to understand which wild pollinators may be beneficial in different locations.

Wild insect species were the most abundant avocado pollinators in many regions and developing approaches that make the most of their contribution may be the most appropriate. This was specifically explored in avocado by two studies identified in the review. Castañeda‐Vildózola et al. (1999) showed that pesticide application reduced the abundance of native pollinators and consequently reduced avocado yields and Villamil et al. (2017) showed that a reduction in intensive orchard management led to an increase in pollinator biodiversity. Similar findings have been observed in many other pollinator reliant crops, and several papers have shown that a reduction in natural habitats or an increase in intensive production leads to a reduction in the abundance and diversity of wild pollinators with negative impacts on yield (Dainese et al., 2019; Garibaldi et al., 2011, 2013; Martin et al., 2019; Reilly et al., 2020). A better understanding of the role of wild pollinators and how management practices can be best adapted to support avocado production is required.

### 4.5 Study limitations

The low number of available studies presented a major limitation. For the meta‐analysis, it was necessary to include papers testing a wide range of variables (fruit set, yield, pollination) and as such, the data were very heterogeneous. Furthermore, some of these studies provided limited information on the methods used, and often, *N* and *SD* were not provided and therefore had to be calculated from the information available. The restricted data availability also meant that statistical analysis was not feasible for the majority of the research questions and therefore, the strength and scope of the conclusions made is limited. However, despite these challenges, some general findings, research gaps and recommendations have been identified.

## 5 CONCLUSIONS

### 5.1 Key findings and research gaps

- Dominican Republic and Mexico are responsible for 37% of global avocado production but only 13% of studies originated from these countries and therefore, further research in these countries is required.
- In 19 out of 23 studies, insect pollinators contributed significantly to pollination, fruit set and yield.
- Managed honeybees were identified as the most important pollinators due to their frequency and efficiency. However, further information is needed to optimize local field beehive placement and density.
- The abundance of wild pollinators ranged from 90% to 8% across locations and further research is required to understand their efficiency and contribution to avocado pollination.
- Land management practices affected the abundance and diversity of wild pollinators and this can have negative implications for yield.

### 5.2 Recommendations for growers

- In most situations, growers will benefit from an increased density of pollinators.
- Increasing honeybee hive density will likely increase production but may not be cost‐effective in all contexts.
- The utilization of alternative managed pollinators (e.g. stingless bees) or active management of wild pollinators may also be applicable in some circumstances.

## CONFLICT OF INTEREST

The authors declare that they have no known competing financial interest or personal relationships that could have appeared to influence the work in this paper.

## AUTHOR CONTRIBUTIONS

Dymond and Garratt conceived research. Dymond conducted systematic review, analysed data, conducted statistical analyses and wrote the manuscript. Garratt supported statistical analyses. All authors provided feedback on the manuscript, read and approved the manuscript.

## Supporting information

Supplementary Material

## References

[jen12869-bib-0001] Afik, O. , Dag, A. , & Shafir, S. (2007). Perception of avocado bloom (Lauraceae: *Persea americana*) by the honey bee (Hymenoptera: Apidae: *Apis mellifera*). Entomologia Generalis, 30, 135–153.

[jen12869-bib-0002] Albrecht, M. , Schmid, B. , Hautier, Y. , & Müller, C. B. (2012). Diverse pollinator communities enhance plant reproductive success. Proceedings of the Royal Society B: Biological Sciences, 279(1748), 4845–4852. 10.1098/rspb.2012.1621 PMC3497085 23034701

[jen12869-bib-0003] Alcaraz, L. M. , & Hormaza, J. I. (2009). Avocado pollination and fruit set – A perspective from Spain. California Avocado Society Yearbook, 92, 113–135.

[jen12869-bib-0004] Alcaraz, M. L. , Hormaza, J. I. , & Rodrigo, J. (2013). Pistil starch reserves at anthesis correlate with final flower fate in avocado (*Persea americana*). PLoS One, 8(10), e78467. 10.1371/journal.pone.0078467 24167627 PMC3805557

[jen12869-bib-0005] Balduzzi, S. , Rücker, G. , & Schwarzer, G. (2019). How to perform a meta‐analysis with R: A practical tutorial. Evidence‐based Mental Health, 22(4), 153–160. 31563865 10.1136/ebmental-2019-300117 PMC10231495

[jen12869-bib-0006] Bergh, B. O. (1967). Reasons for low yields of avocado. California Avocado Society Yearbook, 51, 161–172.

[jen12869-bib-0008] Biesmeijer, J. C. , Roberts, S. P. M. , Reemer, M. , Ohlemüller, R. , Edwards, M. , Peeters, T. , Schaffers, A. P. , Potts, S. G. , Kleukers, R. , & Thomas, C. D. (2006). Parallel declines in pollinators and insect‐pollinated plants in Britain and the Netherlands. Science, 313(5785), 351–354. 10.1126/science.1127863 16857940

[jen12869-bib-0009] Brown, J. , & Cunningham, S. A. (2019). Global‐scale drivers of crop visitor diversity and the historical development of agriculture. Proceedings of the Royal Society B, 286(1915), 20192096. 10.1098/rspb.2019.2096 31744437 PMC6892048

[jen12869-bib-0010] Bushuru, E. (2015). Diversity and pollination activity of flower visiting insect associated with avocado along the slopes of Taita Hills in Kenya. Masters Thesis, Masinde Muliro University of Science and Technology.

[jen12869-bib-0011] Cabezas, C. , & Cuevas, J. (2007). Avocado pollinators in southeast Spain. World Avocado Congress. Chile.

[jen12869-bib-0012] Can‐Alonzo, C. , Quezada‐Euan, J. J. , Xiu‐Ancona, P. , Moo‐Valle, H. , Valdovinos‐Nunez, G. , & Medina‐Peralta, S. (2005). Pollination of ‘criollo’ avocados (*Persea americana*) and the behaviour of associated bees in subtropical Mexico. Journal of Apicultural Research, 44, 3–8. 10.1080/00218839.2005.11101138

[jen12869-bib-0016] Castañeda‐Vildózola, A. , Equihua‐Martínez, A. , Valdés‐Carrasco, J. , Barrientos‐Priego, A. F. , Ish‐Am, G. , & Gazit, S. (1999). Insectos polinizadores del aguacatero en los estados de México y Michoacán. Revista Chapingo Serie Horticultura, 5, 129–136.

[jen12869-bib-0017] Clark, O. I. (1923). Avocado pollination and bees. California Avocado Association Annual Report.

[jen12869-bib-0018] Cook, D. F. , Voss, S. C. , Finch, J. T. D. , Rader, R. C. , Cook, J. M. , & Spurr, C. J. (2020). The role of flies as pollinators of horticultural crops: An Australian case study with worldwide relevance. Insects, 11(6), 341. 10.3390/insects11060341 PMC7349676 32498457

[jen12869-bib-0019] Dafni, A. (1992). Pollination ecology: A practical approach. Oxford University Press.

[jen12869-bib-0020] Dainese, M. , Martin, E. A. , Aizen, M. A. , Albrecht, M. , Bartomeus, I. , Bommarco, R. , Carvalheiro, L. G. , Chaplin‐Kramer, R. , Gagic, V. , Garibaldi, L. A. , Ghazoul, J. , Grab, H. , Jonsson, M. , Karp, D. S. , Kennedy, C. M. , Kleijn, D. , Kremen, C. , Landis, D. A. , Letourneau, D. K. , … Steffan‐Dewenter, I. (2019). A global synthesis reveals biodiversity‐mediated benefits for crop production. Science Advances, 5(10), eaax0121. 10.1126/sciadv.aax0121 31663019 PMC6795509

[jen12869-bib-0021] Davenport, T. L. (2019). Cross‐ vs. self‐pollination in ‘Hass’ avocados growing in coastal and inland orchards of Southern California. Scientia Horticulturae, 246, 307–316. 10.1016/j.scienta.2018.10.051

[jen12869-bib-0022] Davenport, T. L. , Parnitzki, P. , Fricke, S. , & Hughes, M. S. (1994). Evidence and significance of self‐pollination of avocados in Florida. Journal of the American Society for Horticultural Science, 119(6), 1200–1207. 10.21273/JASHS.119.6.1200

[jen12869-bib-0024] Eardley, C. D. , & Mansell, M. W. (1996). The natural occurrence of insect pollinators in an avocado orchard. South African Avocado Growers Association Yearbook, 19, 36–38.

[jen12869-bib-0027] Evans, L. J. , Goodwin, R. M. , & McBrydie, H. M. (2010). Factors affecting Hass avocado (*Persea americana*) fruit set in New Zealand. New Zealand Plant Protection, 63, 214–218. 10.30843/nzpp.2010.63.6548

[jen12869-bib-0028] FAO (2020). FAOSTAT statistical database. FAO.

[jen12869-bib-0029] Fetscher, A. , Davenport, T. , Shafir, S. , Dag, A. , Waser, N. , & Arpaia, M. (2000). A review of avocado pollination and the role of pollinizers. Subtropical Fruit News, 8, 21–25.

[jen12869-bib-0030] Garibaldi, L. A. , Bartomeus, I. , Bommarco, R. , Klein, A. M. , Cunningham, S. A. , Aizen, M. A. , Boreux, V. , Garratt, M. P. D. , Carvalheiro, L. G. , Kremen, C. , Morales, C. L. , Schüepp, C. , Chacoff, N. P. , Freitas, B. M. , Gagic, V. , Holzschuh, A. , Klatt, B. K. , Krewenka, K. M. , Krishnan, S. , … Woyciechowski, M. (2015). Editor's Choice: Review: Trait matching of flower visitors and crops predicts fruit set better than trait diversity. Journal of Applied Ecology, 52(6), 1436–1444. 10.1111/1365-2664.12530

[jen12869-bib-0031] Garibaldi, L. A. , Pérez‐Méndez, N. , Garratt, M. P. D. , Gemmill‐Herren, B. , Miguez, F. E. , & Dicks, L. V. (2019). Policies for ecological intensification of crop production. Trends in Ecology & Evolution, 34(4), 282–286. 10.1016/j.tree.2019.01.003 30745253

[jen12869-bib-0032] Garibaldi, L. A. , Steffan‐Dewenter, I. , Kremen, C. , Morales, J. M. , Bommarco, R. , Cunningham, S. A. , Carvalheiro, L. G. , Chacoff, N. P. , Dudenhöffer, J. H. , Greenleaf, S. S. , Holzschuh, A. , Isaacs, R. , Krewenka, K. , Mandelik, Y. , Mayfield, M. M. , Morandin, L. A. , Potts, S. G. , Ricketts, T. H. , Szentgyörgyi, H. , … Klein, A. M. (2011). Stability of pollination services decreases with isolation from natural areas despite honey bee visits. Ecology Letters, 14(10), 1062–1072. 10.1111/j.1461-0248.2011.01669.x 21806746

[jen12869-bib-0033] Garibaldi, L. A. , Steffan‐Dewenter, I. , Winfree, R. , Aizen, M. A. , Bommarco, R. , Cunningham, S. A. , Kremen, C. , Carvalheiro, L. G. , Harder, L. D. , Afik, O. , Bartomeus, I. , Benjamin, F. , Boreux, V. , Cariveau, D. , Chacoff, N. P. , Dudenhoffer, J. H. , Freitas, B. M. , Ghazoul, J. , Greenleaf, S. , … Klein, A. M. (2013). Wild pollinators enhance fruit set of crops regardless of honey bee abundance. Science, 339(6127), 1608–1611. 10.1126/science.1230200 23449997

[jen12869-bib-0034] Garner, L. C. , & Lovatt, C. J. (2008). The relationship between flower and fruit abscission and alternate bearing of ‘Hass’ avocado. Journal of the American Society for Horticultural Science, 133(1), 3–10. 10.21273/JASHS.133.1.3

[jen12869-bib-0036] Gazit, S. , & Degani, C. (2002). Reproductive biology. In B. A. Schaffer , B. N. Wolstenholme , & A. W. Whiley (Eds.), The avocado: Botany, production and uses (pp. 118–168). CABI.

[jen12869-bib-0037] Goulson, D. (2010). Impacts of non‐native bumblebees in Western Europe and North America. Applied Entomology and Zoology, 45(1), 7–12. 10.1303/aez.2010.7

[jen12869-bib-0038] Howlett, B. G. , & Gee, M. (2019). The potential management of the drone fly (*Eristalis tenax*) as a crop pollinator in New Zealand. New Zealand Plant Protection, 72, 221–230. 10.30843/nzpp.2019.72.304

[jen12869-bib-0039] Howlett, B. G. , Read, S. F. J. , Alavi, M. , Cutting, B. T. , Nelson, W. R. , Goodwin, R. M. , Cross, S. , Thorp, T. G. , & Pattemore, D. E. (2019). Cross‐pollination enhances macadamia yields, even with branch‐level resource limitation. HortScience, 54(4), 609–615. 10.21273/HORTSCI13329-18

[jen12869-bib-0040] Ings, T. C. , Schikora, J. , & Chittka, L. (2005). Bumblebees, humble pollinators or assiduous invaders? A population comparison of foraging performance in *Bombus terrestris* . Oecologia, 144(3), 508–516. 10.1007/s00442-005-0081-9 15891827

[jen12869-bib-0041] Ish‐Am, G. (2005). Avocado pollination – A review. *New Zealand and Australia Avocado Growers' Conference, Tauranga New Zealand, 2005*.

[jen12869-bib-0042] Ish‐Am, G. , Barrientos‐Priego, A. , Castañeda‐Vildózola, A. , & Gazit, S. (1999). Avocado (*Persea americana* Mill.) pollinators in its region of origin. Revista Chapingo Serie Horticultura, 5, 137–143.

[jen12869-bib-0043] Ish‐Am, G. , & Eisikowitch, D. (1990). Possible routs of avocado tree pollination by honeybees. Acta Horticulture, 288, 225–233. 10.17660/ActaHortic.1991.288.33

[jen12869-bib-0044] Ish‐Am, G. , & Eisikowitch, D. (1998). Low attractiveness of avocado (*Persea americana* Mill.) flowers to honeybees (*Apis mellifera* L.) limits fruit set in Israel. Journal of Horticultural Science and Biotechnology, 73(2), 195–204. 10.1080/14620316.1998.11510965

[jen12869-bib-0045] Ish‐Am, G. , & Lahav, E. (2011). Evidence for a major role of honeybees (*Apis mellifera*) rather than wind during avocado (*Persea americana* Mill.) pollination. The Journal of Horticultural Science and Biotechnology, 86(6), 589–594. 10.1080/14620316.2011.11512808

[jen12869-bib-0046] Ish‐Am, G. , Regev, Y. , Degani, C. , & Gazit, S. (1998). Improving avocado pollination with bumblees: 3 Season summary. California Avocado Society Yearbook, 82, 119–135.

[jen12869-bib-0049] Klein, A.‐M. , Vaissiere, B. E. , Cane, J. H. , Steffan‐Dewenter, I. , Cunningham, S. A. , Kremen, C. , & Tscharntke, T. (2007). Importance of pollinators in changing landscapes for world crops. Proceedings of the Royal Society B: Biological Sciences, 274(1608), 303–313. 10.1098/rspb.2006.3721 PMC1702377 17164193

[jen12869-bib-0050] Kremer‐Köhne, S. , & Köhne, J. S. (1995). Approaches to solving the Hass small fruit problem: Progress report. South African Avocado Growers' Association Yearbook, 18, 59–60.

[jen12869-bib-0051] Lesley, J. W. , & Bringhurst, R. S. (1951). Environmental conditions affecting pollination of avocados. California Avocado Society Yearbook, 35, 169–173.

[jen12869-bib-0052] Magrach, A. , & Sanz, M. J. (2020). Environmental and social consequences of the increase in the demand for ‘superfoods’ world‐wide. People and Nature, 2(2), 267–278. 10.1002/pan3.10085

[jen12869-bib-0053] Malerbo‐Souza, D. , Arnaut de Toledo, V. , Silva, S. , & Sousa, F. (2000). Polinização em flores de abacateiro (*Persea americana* Mill.). Acta Scientiarum Agronomy, 22, 937–941. 10.4025/actasciagron.v22i0.2841

[jen12869-bib-0054] Martin, E. A. , Dainese, M. , Clough, Y. , Báldi, A. , Bommarco, R. , Gagic, V. , Garratt, M. P. D. , Holzschuh, A. , Kleijn, D. , Kovács‐Hostyánszki, A. , Marini, L. , Potts, S. G. , Smith, H. G. , Al Hassan, D. , Albrecht, M. , Andersson, G. K. S. , Asís, J. D. , Aviron, S. , Balzan, M. V. , … Steffan‐Dewenter, I. (2019). The interplay of landscape composition and configuration: New pathways to manage functional biodiversity and agroecosystem services across Europe. Ecology Letters, 22(7), 1083–1094. 10.1111/ele.13265 30957401

[jen12869-bib-0056] McNeil, R. , & Pidduck, W. (2003). The effectiveness of the western bumblebee in pollinating Hass avocado trees. World Avocado Congress.

[jen12869-bib-0060] Mulwa, J. , Kahuthia‐Gathu, R. , & Kasina, M. (2019). Avocado (*Persea americana*) yield as influnced by pollinators in Murang'a County. Kenya Agricultural Research Advances, 01, 34–41.

[jen12869-bib-0061] Nirody, B. (1922). Investigations in avocado breeding. Master of Science, Massachusetts Agricultural College.

[jen12869-bib-0062] Papademetriou, M. K. (1976). Some aspects of the flower behavior, pollination and fruit set of avocado (*Persea americana* Mill.) in Trinidad. California Avocado Society, 60, 106–152.

[jen12869-bib-0063] Pattemore, D. , Buxton, M. N. , Cutting, B. T. , McBrydie, H. , Goodwin, M. , & Dag, A. (2018). Low overnight temperature associated with a delay in ‘Hass’ Avocado (*Persea americana*) female flower opening, leading to nocturnal flowering. *Journal of Pollination* . Ecology, 23. 10.26786/1920-7603%282018%2912

[jen12869-bib-0064] Peña, J. F. , & Carabalí, A. (2018). Effect of honey bee (*Apis mellifera* L.) density on pollination and fruit set of avocado (*Persea americana* Mill.). Cv. Hass. Journal of Apicultural Science, 62(1), 5–14. 10.2478/jas-2018-0001

[jen12869-bib-0065] Perez‐Balam, J. , Quezada‐Euan, J. J. , Alfaro‐Bates, R. , Medina, S. , McKendrick, L. , Soro, A. , & Paxton, R. (2012). The contribution of honey bees, flies and wasps to avocado (*Persea americana*) pollination in southern Mexico. Journal of Pollination Ecology, 8, 42–47. 10.26786/1920-7603%282012%296

[jen12869-bib-0066] Petersen, P. A. (1955). Avocado flower pollination and fruit set. California Avocado Society, 39, 163–169.

[jen12869-bib-0067] Potts, S. G. , Imperatriz‐Fonseca, V. , Ngo, H. T. , Aizen, M. A. , Biesmeijer, J. C. , Breeze, T. D. , Dicks, L. V. , Garibaldi, L. A. , Hill, R. , Settele, J. , & Vanbergen, A. J. (2016). Safeguarding pollinators and their values to human well‐being. Nature, 540(7632), 220–229. 10.1038/nature20588 27894123

[jen12869-bib-0068] Quezada‐Euán, J. J. G. , de Jesús May‐Itzá, W. , & González‐Acereto, J. A. (2001). Meliponiculture in Mexico: Problems and perspective for development. Bee World, 82(4), 160–167. 10.1080/0005772X.2001.11099523

[jen12869-bib-0069] R Core Team (2019). A language and environment for statistical computing. R Foundation for Statistical Computing. http://www.R‐project.org/

[jen12869-bib-0070] Rader, R. , Cunningham, S. A. , Howlett, B. G. , & Inouye, D. W. (2020). Non‐bee insects as visitors and pollinators of crops: Biology, ecology, and management. Annual Review of Entomology, 65, 391–407. 10.1146/annurev-ento-011019-025055 31610136

[jen12869-bib-0071] Ramírez, V. M. , Ayala, R. , & González, H. D. (2018). Crop pollination by stingless bees. In P. Vit S. R.M. Pedro & D. W. Roubik (Eds.), Pot‐pollen in stingless bee melittology (pp. 193–153). Springer. 10.1007/978-3-319-61839-5_11

[jen12869-bib-0073] Reilly, J. R. , Artz, D. R. , Biddinger, D. , Bobiwash, K. , Boyle, N. K. , Brittain, C. , Brokaw, J. , Campbell, J. W. , Daniels, J. , Elle, E. , Ellis, J. D. , Fleischer, S. J. , Gibbs, J. , Gillespie, R. L. , Gundersen, K. B. , Gut, L. , Hoffman, G. , Joshi, N. , Lundin, O. , … Winfree, R. (2020). Crop production in the USA is frequently limited by a lack of pollinators. Proceedings of the Royal Society B, 287(1931), 20200922. 10.1098/rspb.2020.0922 33043867 PMC7423660

[jen12869-bib-0075] Robbertse, P. , & Johannsmeier, M. (1997). Pollination studies in Hass avocado in relation to the small fruit problem. Journal of Systems Architecture – JSA, 20, 84–85.

[jen12869-bib-0076] Rollin, O. , & Garibaldi, L. A. (2019). Impacts of honeybee density on crop yield: A meta‐analysis. Journal of Applied Ecology, 56(5), 1152–1163. 10.1111/1365-2664.13355

[jen12869-bib-0077] Sedgley, M. (1977). Reduced pollen tube growth and the presence of callose in the pistil of the male floral stage of the avocado. Scientia Horticulturae, 7(1), 27–36. 10.1016/0304-4238(77)90040-1

[jen12869-bib-0078] Sedgley, M. , & Grant, W. J. R. (1983). Effect of low temperatures during flowering on floral cycle and pollen tube growth in nine avocado cultivars. Scientia Horticulturae, 18(3), 207–213. 10.1016/0304-4238(83)90023-7

[jen12869-bib-0079] Sedgley, M. , & Griffin, A. R. (1989). Sexual reproduction of tree crops. Academic Press.

[jen12869-bib-0080] Slaa, E. J. , Chaves, L. A. S. , Malagodi‐Braga, K. S. , & Hofstede, F. E. (2006). Stingless bees in applied pollination: Practice and perspectives. Apidologie, 37(2), 293–315. 10.1051/apido:2006022

[jen12869-bib-0081] Stout, A. B. (1932). Sex in avocados and pollination. California Avocado Association, 17, 172–173.

[jen12869-bib-0082] Villamil, L. , Astier, M. , Merlín‐Uribe, Y. , Ayala, R. , Ramírez‐García, E. , Cruz, J. , Devoto, M. , & Gavito, M. (2017). Management practices and diversity of flower visitors and herbaceous plants in conventional and organic avocado orchards in Michoacán, Mexico. Agroecology and Sustainable Food Systems, 45, 530–551. 10.1080/21683565.2017.1410874

[jen12869-bib-0084] Vithanage, V. (1990). The role of the European honeybee (*Apis mellifera* L.) in avocado pollination. Journal of Horticultural Science, 65(1), 81–86. 10.1080/00221589.1990.11516033

[jen12869-bib-0085] Webber, S. M. , Garratt, M. P. D. , Lukac, M. , Bailey, A. P. , Huxley, T. , & Potts, S. G. (2020). Quantifying crop pollinator‐dependence and pollination deficits: The effects of experimental scale on yield and quality assessments. Agriculture, Ecosystems & Environment, 304, 107106. 10.1016/j.agee.2020.107106

[jen12869-bib-0086] Wickham, H. (2016). ggplot2: Elegant graphics for data analysis. Springer.

[jen12869-bib-0088] Woodcock, B. A. , Garratt, M. P. D. , Powney, G. D. , Shaw, R. F. , Osborne, J. L. , Soroka, J. , Lindström, S. A. M. , Stanley, D. , Ouvrard, P. , & Edwards, M. E. (2019). Meta‐analysis reveals that pollinator functional diversity and abundance enhance crop pollination and yield. Nature Communications, 10(1), 1–10. 10.1038/s41467-019-09393-6 PMC6443707 30931943

[jen12869-bib-0089] Wysoki, M. , Van den Berg, M. , Ish‐Am, G. , Gazit, S. , Peña, J. , & Waite, G. (2002). Pest and pollinators of avocado. In J. Peña , J. Sharp , & M. Wysoki (Eds.), Tropical fruit pest and pollinators (pp. 223–295). CABI.

[jen12869-bib-0090] Ying, Z. , Davenport, T. L. , Faber, B. , Zhang, T. , Schnell, R. J. , & Tondo, C. L. (2009). Re‐evaluation of the roles of honeybees and wind on pollination in avocado. The Journal of Horticultural Science and Biotechnology, 84(3), 255–260. 10.1080/14620316.2009.11512513

