# Supplementary material for: The role of insect pollinators in avocado production: A global review
Source: J Appl Entomol. 2021 Feb 9;145(5):369–83. doi: 10.1111/jen.12869 (PMC8647928; doi:10.1111/jen.12869)
Supplement: Supplementary file 1 — Supplementary Material [file JEN-145-369-s001.docx]

**Supplementary Information**

**SI Table 1:** Summary of papers used in the review.

| **Study** | **Location** | **Variable Studied** | **Main Finding** | **Research Aim** |
| --- | --- | --- | --- | --- |
| Davenport  (2019) | USA | The contribution to pollination from pollinators and wind | Most pollination took place in stage 2 and this was primarily self-pollination. Wind contributed more than pollinators. This was shown in humid and dry environments. | Effect of insect pollinators on pollination and yield |
| Pena and Carabali  (2018) | Colombia | The effect of honey bee density on yield | Higher honeybee density led to higher fruit set. Distance from the hives did not influence fruit set. Honey bees were more efficient pollinators than other species. | Effect of insect pollinators on pollination and yield |
| Ish-Am and Lahav  (2011) | Israel | Contribution to pollination from pollinators and wind | Bee density was positively correlated with fruit set and yield. Wind did not contribute much to pollination. | Effect of insect pollinators on pollination and yield |
| Davenport *et al., (1994)* | USA | Contribution to pollination from pollinators and wind | There was no significant difference in the rate of pollination for open and closed treatments. Most pollination is self- pollination during stage 2. | Effect of insect pollinators on pollination and yield |
| Malerbo-Souza *et al.,*  *(2000)* | Brazil | Contribution to pollination from pollinators | Crop yield was reduced by 81% without pollinators. Open pollination produced 2.5 fruits per branch and closed 0.5 fruits per branch. | Effect of insect pollinators on pollination and yield |
| Johannsmeier et al., (1997) | South Africa | Contribution to pollination from honeybees and polliniser trees | Closed treatments and treatments without polliniser trees had significantly lower yields than open treatments and treatments with polliniser trees | Effect of insect pollinators on pollination and yield |
| Mulwa *et al.,(2019a)* | Kenya | Contribution to pollination from pollinators and the abundance and variety of pollinators | Pollinators lead to a 330% increase in yield. The major pollinators observed were: honey bees, wasps, drone fly and blow fly. | Effect of insect pollinators on pollination and yield and pollinator abundance |
| Bezuidenhout et al.,(2016) | South Africa | Contribution to pollination from pollinators and different pollinizer trees | Honeybees led to significantly higher yields. Did see an increase with polliniser trees, but not clear if it is cost effective. | Effect of insect pollinators on pollination and yield |
| Cabezas and Cuevas,  (2007) | Spain | Contribution to pollination from pollinators and wind. | Honeybees and bumblebees significantly increased fruit set. Wind was not significant. | Effect of insect pollinators on pollination and yield |
| Peterson, (1955) | USA | Contribution to pollination from pollinators | There was a significantly higher yield in cages with bees and very limited fruit set in cages with no bees. | Effect of insect pollinators on pollination and yield |
| Alcaraz and Hormaza, (2009) | Spain | Yield limitations due to pollination and contribution to pollination from pollinizer trees | Hand pollination was significantly higher than open pollination. Most of the final fruits were the result of outcrossing. Different pollinizer trees were more effective than others. | Effect of insect pollinators on pollination and yield |
| Robbertse *et al.,(1996)* | South Africa | Contribution to pollination from pollinators and pollinizer trees | Open pollinated treatments had significantly higher fruit set than closed treatments. Etinger pollen contributed significantly. Distance from polliniser tree showed mixed results across years | Effect of insect pollinators on pollination and yield |
| Gazit, 1977 |  | Contribution to pollination from pollinators and polliniser trees | Self-pollination can occur but yields increase with bees and polliniser trees | Effect of insect pollinators on pollination and yield |
| Carabalí-Banguero *et al.*, 2018b | Colombia | Contribution to pollination from pollinators | Fruit set was significantly higher in open pollinated treatments in comparison to closed treatments | Effect of insect pollinators on pollination and yield |
| Johannsmeier and Morudu, 1999 | South Africa | Contribution to pollination from pollinators and polliniser trees | Open pollinated treatments had significantly higher fruit set than closed treatments. Fruit drop was less when cultivars were cross pollinated but single cultivar pollination still yielded well. | Effect of insect pollinators on pollination and yield |
| Vithanage (1990) | Australia | Abundance and effectiveness of different avocado pollinators. Contribution to yield from different bee hive densities | A wide variety of insects contributed to pollination but honeybees were the most important. Yields significantly increased when hives were added and fruit size increased at high beehive density. | Effect of insect pollinators on pollination and yield |
| Materu, 2019 | Tanzania | Abundance of pollinators | Honeybees were the most abundant (60%) but several other wild pollinator species were also observed, with stingless bees being the second most abundant species (26%) | Pollinator abundance |
| Willcox *et al*., (2019) | Australia | Abundance and effectiveness of pollinators contributing to 3 key crops, including avocado | A variety of species were present on avocado flowers but most common were Apis Mellifera, Tetragon ula spp and Stomarhina discolor. Apis Mellifera was the main contributor. | Pollinator abundance |
| Carabalí-Banguero *et al.*, 2018a | Colombia | Abundance of pollinators | Honeybees were the main pollinators but a high diversity of other species was observed especially in the Diptera order. | Pollinator abundance |
| Estévez and Martínez, 2020 | Cuba | Abundance and efficiency of pollinators | Honeybees were the most abundant species and Diptera was the most abundant order. Dipterans were potentially effective pollinators as they made contact with the reproductive organs of the flowers. | Pollinator abundance |
| Read *et al.,* (2017) | New Zealand | Abundance of pollinators | Honey bees were the most abundant but there was a high variation in different sites. Beetles and bumblebees were also commonly observed. | Pollinator abundance |
| Eardley and Mansell (1996) | South Africa | Abundance of pollinators | The honeybee was the most abundant and significant pollinator. Other species were effective but had low abundance. | Pollinator abundance |
| Mulwa *et al.,(2019b)* | Kenya | Abundance of pollinators | Honey bees, blow flies, hoverflies and wasps were the major avocado flower visitor. | Pollinator abundance |
| Ish-a *et al.,(1999)* | Mexico | Abundance and effectiveness of avocado pollinators | Honeybees were the main pollinators but there were many native species that contributed. | Pollinator abundance and effectiveness |
| Bushuru  (2015) | Kenya | Abundance and effectiveness of avocado pollinators | A range of insects were found on avocado flowers but honeybees were the most efficient as they carried the most pollen and were the most abundant. | Pollinator abundance and effectiveness |
| Mehmood *et al*  (2015) | Pakistan | Abundance of pollinator | Hymenopteran pollinators were higher in numbers (42%) followed by Lepidopterans (33%) and Dipterans (25%) | Pollinator abundance |
| Can-Alonzo et al., (2005) | Mexico | Abundance and effectiveness of avocado pollinators | Stingless and honeybees are the most efficient pollinators. Flies and wasps were also observed but these were not as abundant. No self-pollination occurred. | Pollinator abundance and effectiveness |
| Perez-Balam *et al* (2012) | Mexico | Abundance and effectiveness of avocado pollinators | Honeybees and flies were more effective pollinators for avocados than wasps. All deposited a similar amount of pollen but honeybees and flies were more abundant. | Pollinator abundance and effectiveness |
| Visscher (1997) | USA | Abundance of pollinators | Honeybees were the most abundant pollinators but wild bees were also observed. | Pollinator abundance |
| Evans *et al*., (2011) | New Zealand and Australia | Abundance of pollinators | Range of pollinators in Australia but not so diverse in New Zealand. The amount of pollen deposition and fertilisation was low in both countries. | Pollinator abundance |
| De la Cuadra (2007) | Chile | Abundance and efficiency of pollinators | Range of pollinators observed, but only a few contributed. Honeybees contributed the most. | Pollinator abundance and effectiveness |
| Castaneda- Vildozola *et al (1999)* | Mexico | Abundance and efficiency of pollinators | Honeybees were the main pollinators but there were many native species that contributed. Biodiversity of pollinators was reduced in areas that were sprayed with pesticides. | Pollinator abundance and effectiveness |
| Carabali-Banguero *et al.,(2020)* | Colombia | Abundance of pollinators and analysis of pollen loads | Several different pollinators were observed to carry avocado pollen. All species also carried pollen from different botanical families. | Pollinator abundance |
| Monzon *et al., (2020)* | Chile | Abundance and efficiency of pollinators | In total 6 pollinator species were observed but the honeybee was the most abundant. Honeybees and wild bees had similar visitation rates. | Pollinator abundance and effectiveness |
| Villamil *et al*., (2017) | Mexico | Impact on diversity of pollinators from landscape and land management. | Intensive management influenced the variety for flowers and pollinator biodiversity. The key factors were insecticides, removal of weeds and forested areas. | Ways to improve insect pollination |
| Afik *et al.,*  (2007) | Israel | Exploring honeybees behaviour around the avocado bloom and reasons for avoidance | High levels of mineral contents (Potassium and Phosphate) present in nectar deter honeybees. Different races and breeds of bees do have different preferences for these minerals. | Ways to improve insect pollination |
| Ish-Am *et al*.,  (1998) | Israel | Efficiency of bumblebees and honeybees as avocado pollinators | In Etinger, Bumblebees increased cross pollination and significantly increased yields. In Hass there was a slight increase in yields mostly due to increases in cross pollination in trees far from hives. | Ways to improve insect pollination |
| Ish-Am and Eisikowitch (1998) | Israel | The contribution and effectiveness of honeybees in avocado pollination. | During late blooming cultivars, fewer non avocado flowers were blooming and therefore avocado flowers received more pollinators and had a higher fruit set in comparison to early blooming cultivars. There was a negative correlation between bee density and pollination. | Ways to improve insect pollination |
| Fetscher *et al.,(2000)* | Israel and USA | Contribution from different races of honey bees and different pollinizer trees to avocado yield. | Results were not significant but suggest that New World Carniolan (NWC) race of bees might be more effective than Italian honeybees as they carried more pollen back to the hive and visited more flowers. Yield increased with closeness to polliniser tree. | Ways to improve insect pollination |
| McNeil and Pidduck (2003) | USA | Effectiveness of Bumblebees as avocado pollinators | Bumblebee, honeybees and syrphid flies were all observed on the flowers. Yields increased in rows closest to bumblebee hives.. | Ways to improve insect pollination |

**SI Table 2:** Calculations of SD and Mean used in the meta-analysis

**SI Table 3:** Raw data used in the meta-analysis (full model).

**SI Table 4:** Raw data used in the meta-analysis (reduced model).

**SI Table 5:** Raw data used to calculate the amount of pollen carried by different pollinators.

**SI Table 6:** Raw data used to calculate the amount of pollen deposited per visit

**SI Table 7:** Raw data used to calculate the number of flowers visited per min.

**A)**


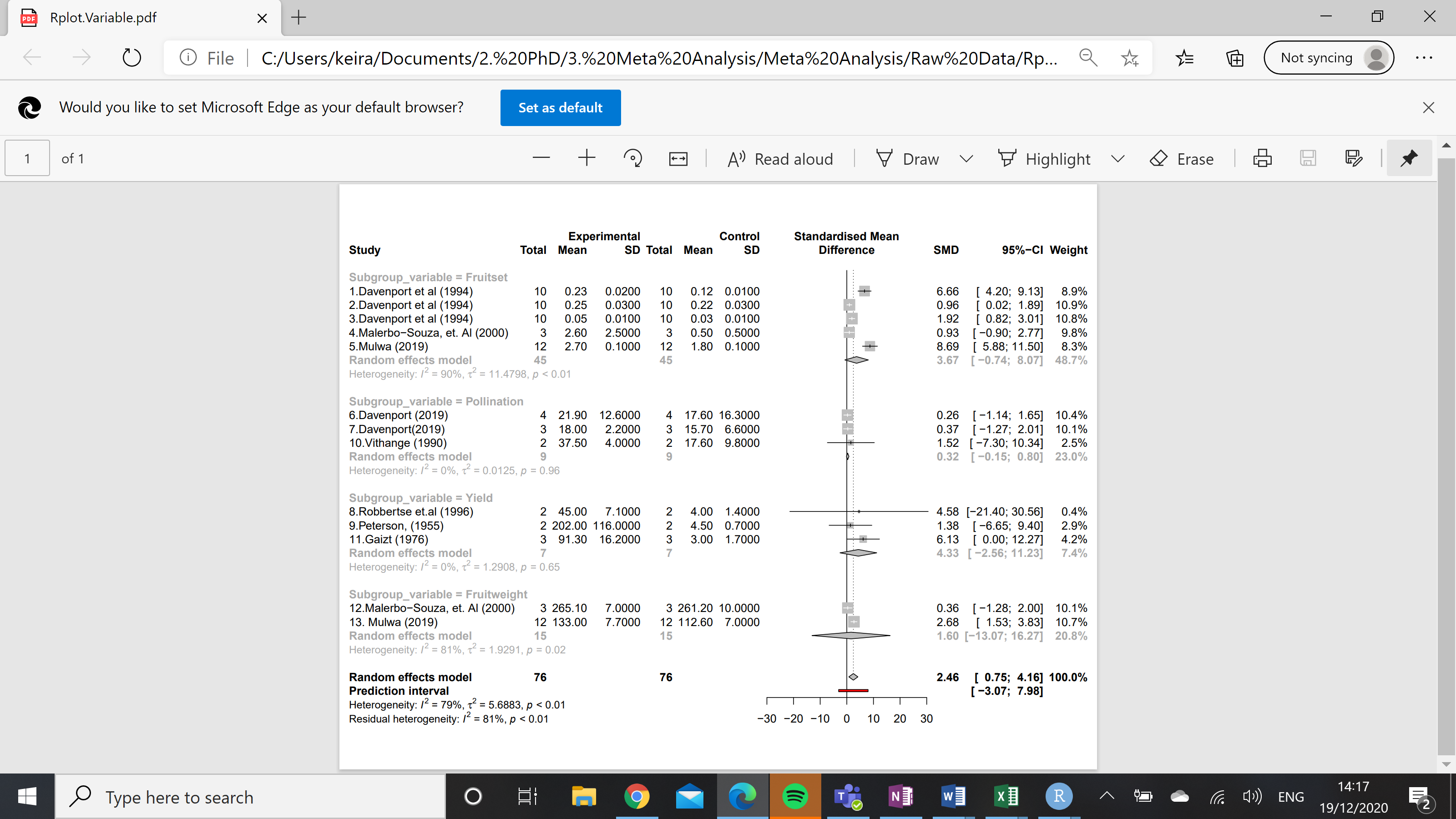


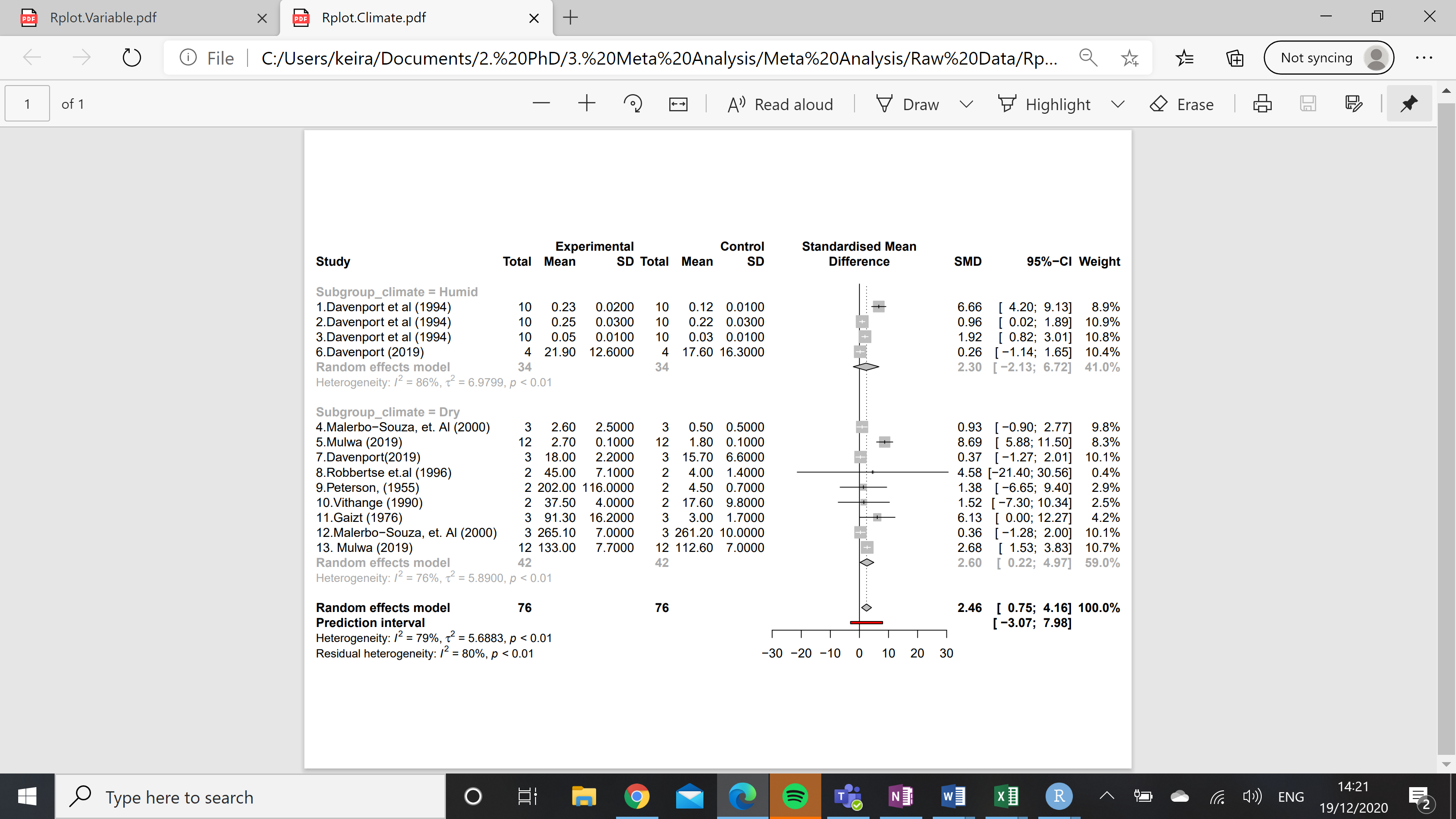
**B)**


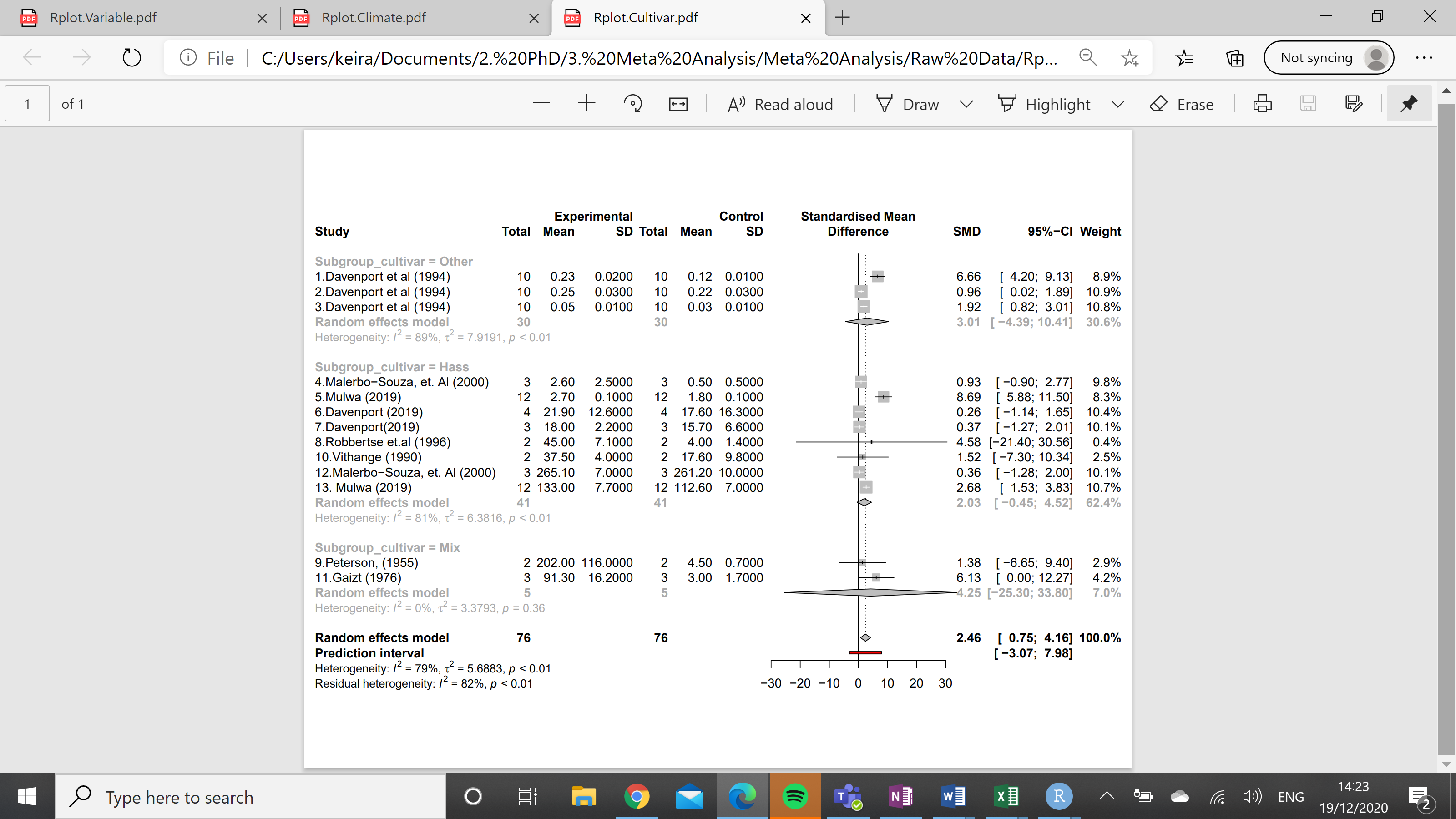
**C)**


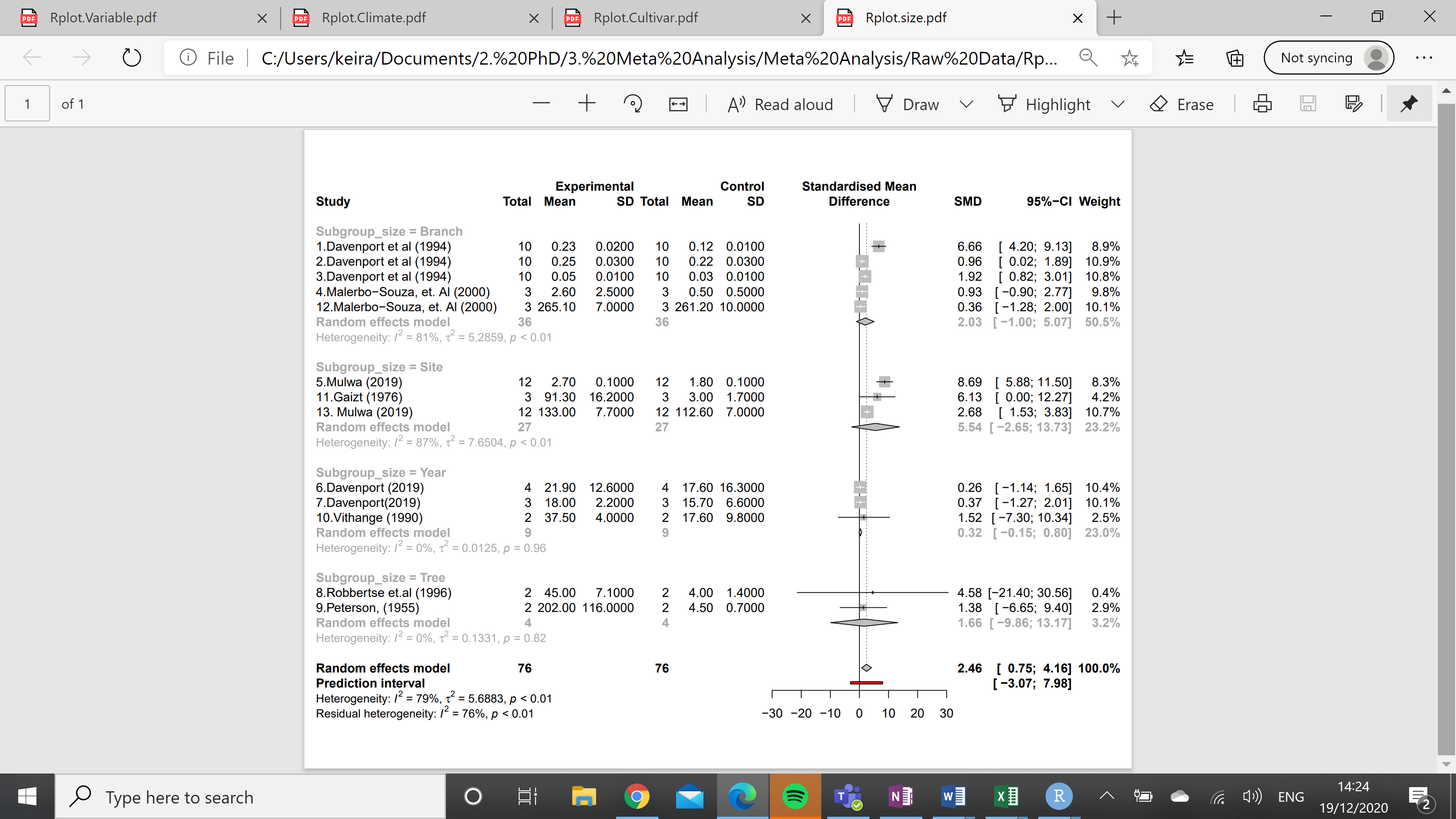
**D)**

**SI Fig 1:** Forest plot following a randomised meta-analysis to compare effects of pollination and production under open (Experimental) and closed (Control) pollination treatments in avocado across multiple studies subcategorised by (A) variable, (B) climate, (C) cultivar and (D) experimental scale.


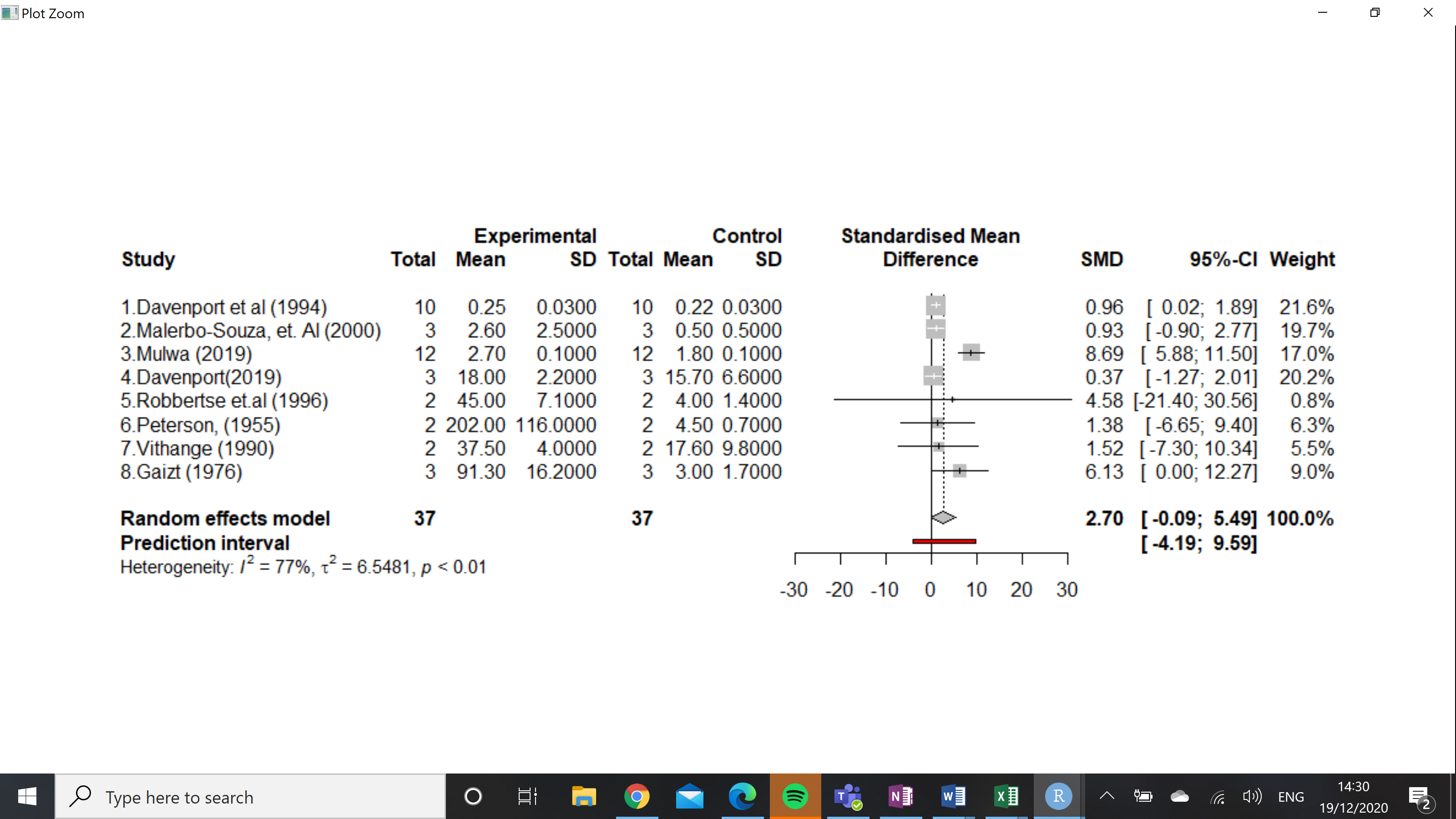


**SI Fig 2:** Forest plot following a randomised meta-analysis to compare effects of pollination and production under open (Experimental) and closed (Control) pollination treatments in avocado using reduced dataset to account for study interdependency.

**References:**

Bezuidenhout, M. M., Du Toit, E. S., & Robbertse, P. J. (2016). Finding the best polliniser for ‘Hass’ avocado and the effect of honeybees as pollinators. South African Avocado Growers Association Yearbook, 40, 70–75.

Carabalí‐Banguero, D., Montoya‐Lerma, J., & Carabalí‐Muñoz, A. (2018a). Dípteros asociados a la floración del aguacate Persea americana Mill cv. Hass en Cauca, Colombia. Biota Colombiana , 19, 92–111.

Carabalí‐Banguero, D., Montoya‐Lerma, J., & Carabalí‐Muñoz, A. (2018b). Efecto de la exclusión de insectos visitantes florales en el cuajado de frutos de Persea americana (Lauraceae) cv. Hass. Acta Zoológica Mexicana, 34, 1–9.

Carabalí‐Banguero, D., Montoya‐Lerma, J., & Carabalí‐Muñoz, A. (2020). Pollen loads on entomofauna visiting flowers of Persea americana (Lauraceae) cv. Hass. Caldasia, 42(1), 105–114. https://doi.org/10.15446/caldasia.v42n1.77136

De la Cuadra, S. (2007). Determination of the pollination activity of honeybees (Apis Mellifera ) in the avocado tree pollination in the Central zone in Chile. World Avocado Congress. Chile.

Estévez, A. A., & Martínez, A. G. (2020). Visitantes florales del aguacate (Persea americana Mill.) en un terreno urbano en La Habana, Cuba. Acta Botánica Cubana, 14–19.

Evans, L. J., Goodwin, M. R., & Howlett, B. (2011). The role of insect pollinators in avocado (Persea americana) pollination in New Zealand and Australia. World Avocado Congress. Australia.

Gazit, S. (1977). Pollination and fruit set of avocado. In Proc., First International Tropical. Fruit Short Course: The Avocado, University of Florida, Gainesville, FL (pp. 88–92).

Johannsmeier, M. F., & Morudu, T. M. (1999). Some factors affecting pollination and yield of Hass avocados. South African Avocado Growers's Association, 22, 22–25.

Johannsmeier, M., Swart, D. J., & Mordud, T. M. (1997). Honeybees in an avocado orchard: Forager distribution, influence on fruit set and colony development. South African Avocado Growers Association Yearbook, 20, 39–41.

Materu, C. L. (2019). Insect pollinators diversity in Avocado orchard during flowering period in Lushoto district Tanzania. International Journal of Research‐Granthaalayah, 7, 20–24.

Mehmood, K., Hussain, S., Mustafa, N., Bodlah, I., & Ahmad, M. (2015). Insect pollinators visiting citrus (Citrus limon) and avocado (Persea americana ) fruit trees. Asian Journal of Agriculture Biology, 3, 23–27.

Monzón, V. H., Avendaño‐Soto, P., Araujo, R. O., Garrido, R., & Mesquita‐Neto, J. N. (2020). Avocado crops as a floral resource for native bees of Chile. Revista Chilena de Historia Natural, 93(1), 1–7. https://doi.org/10.1186/s40693‐020‐00092‐x

Mulwa, J. M., Gathu, R. K., Matolo, N., Guantai, M. M., & Kasina, J. M. (2019). Avocado (Persea americana ) floral calendar and diurnal visitation rates of its pollinators in Murang'a Kenya. Agricultural Research Advances, 1, 23–27.

Read, S., Howlett, B., Jesson, L. K., & Pattemore, D. (2017). Insect visitors to avocado flowers in the Bay of Plenty, New Zealand. New Zealand Plant Protection, 70, 38–44. https://doi.org/10.30843/nzpp.2017.70.25

Robbertse, P., Coetzer, L., Johannsmeier, M., Swart, D., & Köhne, S. (1996). Hass yield and fruit size as influenced by pollination and pollen donor – A joint progress report. Journal of Systems Architecture – JSA, 19, 63–67.

Visscher, P. K. (1997). Avocado pollination in California growing conditions. In California Avocado Research Symposium.

Willcox, B., Howlett, B., Robson, A., Cutting, B., Evans, L., Jesson, L., Kirkland, L., Jm, M., Potdevin, V., Saunders, M., & Rader, R. (2019). Evaluating the taxa that provide shared pollination services across multiple crops and regions. Scientific Reports, 9, 1–10. https://doi.org/10.1038/s41598‐019‐49535‐w
